# Drug‐induced liver injury by glatiramer acetate leading to liver transplant: A case report

**DOI:** 10.1002/jgh3.12938

**Published:** 2023-07-26

**Authors:** Nikan Amirkhani, Amirmohammad Khalaji, Parsa Alehossein, Masoomeh Safaei, Najme Aletaha, Arash Miroliaee

**Affiliations:** ^1^ School of Medicine Tehran University of Medical Sciences Tehran Iran; ^2^ Non‐communicable Diseases Research Center, Endocrinology and Metabolism Population Sciences Institute Tehran University of Medical Sciences Tehran Iran; ^3^ Neuroscience Research Center Shahid Beheshti University of Medical Sciences Tehran Iran; ^4^ Department of Pathology, Cancer Institute, Imam Khomeini Hospital Complex Tehran University of Medical Sciences Tehran Iran; ^5^ Division of Gastroenterology and Hepatology, Imam Khomeini Hospital Complex Tehran University of Medical Sciences Tehran Iran

**Keywords:** drug‐induced liver injury, glatiramer acetate, liver failure, liver transplantation, multiple sclerosis

## Abstract

Glatiramer acetate (GA) is a widely used immune‐modulating drug in relapsing multiple sclerosis (MS). Although a few cases of drug‐induced liver injury during GA therapy have been reported earlier, herein we present the case of a 43‐year‐old woman with relapsing MS who experienced acute liver failure after GA therapy, ultimately leading to liver transplant.

## Graphical abstract

This case report involves a 43‐year‐old woman with relapsing multiple sclerosis (MS) who experienced acute liver failure after glatiramer acetate (GA) therapy, ultimately leading to a liver transplant. GA is a widely used immune‐modulating drug in relapsing MS.

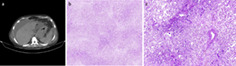

## Introduction

Multiple sclerosis (MS) is a chronic inflammatory disorder of the central nervous system (CNS) characterized by autoimmune lymphocytic infiltration. This leads to focal areas of demyelination, inflammation, and glial reaction in the white matter. Clinical presentation of MS involves repetitive episodes of neurological disability, which may lead to persistent mobility and cognitive dysfunction.^1^, ^2^ There is no recognized curative treatment for MS. However, treatment with disease‐modifying therapies (DMTs) appears to reduce the frequency of relapses and disability progression.^3^, ^4^, ^5^

Glatiramer acetate (GA) is a synthetic polypeptide that antigenically resembles the myelin basic protein (MBP). GA has been prescribed as a first‐line DMT in relapse‐remitting MS over the last two decades. Evidence suggests that GA administration promotes the immunomodulation of both innate and adaptive immune systems. Furthermore, GA exerts some neuroprotective and remyelinating effects.^6^, ^7^ GA is considered a safe and well‐tolerated treatment,^8^ with no prerequisite blood tests for therapy monitoring.^9^ However, a relatively rare but important side effect of GA is liver damage, with 12 reported cases in the literature.^10^ This article presents a case of drug‐induced liver injury (DILI) caused by GA, which led to a liver transplant.

## Case report

A 43‐year‐old woman with a known case of MS was referred to Imam Khomeini Hospital Complex, Tehran, Iran, with a diagnosis of DILI following GA therapy. The patient presented with abdominal pain, jaundice, nausea/vomiting, abnormal liver function tests (LFT), and impaired coagulation assays. The patient had been diagnosed with MS 7 months ago and was prescribed GA 4 months later due to the ineffectiveness of corticosteroid therapy. She was prescribed subcutaneous GA 20 mg three times a week for 6 weeks. The patient developed signs of acute hepatotoxicity in the last week of treatment, leading to ward admission in Hamedan, Iran, and the discontinuation of GA. Given the progressive worsening of the patient's symptoms and lab findings, she was referred to our center with a possible liver failure diagnosis as a liver transplant case. The patient had no other relevant past medical or drug history. She does not smoke or drink alcohol and denies using herbal remedies. Her physical examination revealed a fever (*T* = 38°C), tachycardia (pulse rate = 110 beats/min), pale conjunctiva and icteric sclera, right upper quadrant abdominal tenderness, jaundice, and diffuse ascites. There was no asterixis or signs of encephalopathy; Murphy's sign was negative.

Pertinent laboratory results are presented in Table 1. Additionally, testing during previous hospital admission had revealed negative serologic markers for hepatitis A, B, and C, as well as negative markers of autoimmunity including antinuclear antibody, anti‐smooth‐muscle antibody, and normal levels of immunoglobulin G (760 mg/dL, normal range: 600–1600). Ceruloplasmin levels were within normal range. Previous lab values indicated no hepatic impairment during or after corticosteroid therapy before initiating GA.

**Table 1:** Initial laboratory results

Test	Level	Normal range
WBC	14.5 (×10^9^/L)	4.5–11
RBC	3.59 (×10^12^/L)	3.8–5.2
Hemoglobin	9.2 (g/dL)	11.6–15
Hematocrit	29.4 (%)	36–48
MCV	81.9 (fL)	80–100
MCH	25.6 (fL)	27–31
MCHC	31.3 (fL)	32–36
Platelets	176 (×1000/L)	150–450
RDW‐SD	71.1 (fL)	40–55
RDW‐CV	25.6	11–15
PDW	14.8 (fL)	9–17
MPV	10.2 (fL)	6.5–12
Sodium	136 (mEq/L)	135–145
Potassium	3.8 (mEq/L)	3.5–5.0
Urea	27 (mg/dL)	15–50
Creatinine	0.9 (mg/dL)	0.7–1.4
AST	143 (unit/L)	<31
ALT	71 (unit/L)	<31
Total bilirubin	28.6 (mg/dL)	0.1–1.2
Direct bilirubin	26.6 (mg/dL)	<0.3
LDH	482 (unit/L)	<480
Amylase	40 (unit/L)	<100
Lipase	60 (unit/L)	<60
ESR	78 (mm/h)	<29
CRP	28 (mg/dL)	<6
PT	18.4 (s)	11–15
INR	1.53	1–1.2
PTT	36 (s)	25–40
Albumin	2.2 (g/dL)	3.5–5.2
Total protein	4.4	6–7.8

ALT, alanine transaminase; AST, aspartate aminotransferase; CRP, C‐reactive protein; ESR, erythrocyte sedimentation rate; INR, international normalized ratio; LDH, lactate dehydrogenase; MCH, mean corpuscular hemoglobin; MCHC, mean corpuscular hemoglobin concentration; MCV, mean corpuscular volume; MPV, mean platelet volume; PDW, platelet distribution width; PT, prothrombin time; PTT, activated partial thromboplastin time; WBC, white blood cells; RBC, red blood cells; RDW‐CV, red cell distribution width—coefficient of variation.

**Figure 1:**
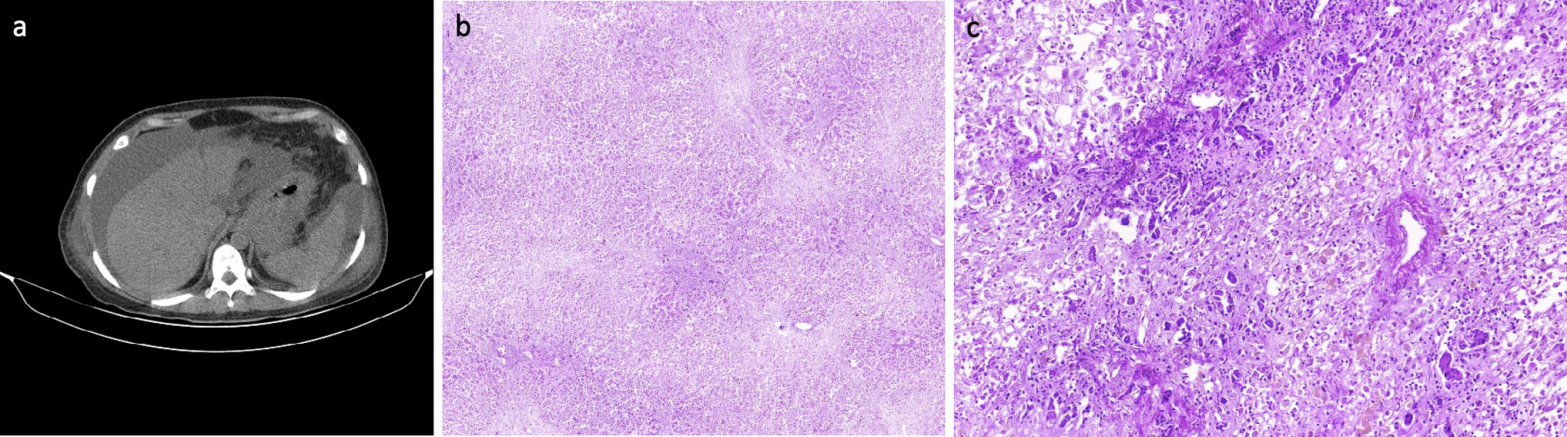
(a) Axial view of abdominal computed tomography scan; (b, c) HE‐stained slides from the whole‐organ excised liver.

An abdominopelvic ultrasound showed a normal‐sized liver with coarse parenchymal echo, normal intrahepatic ducts and no splenic or pancreatic abnormalities. Severe ascites was observed. A spiral abdominopelvic computed tomography (CT) scan revealed severe gallbladder wall thickening and pericholecystic edema as well as edema of the intrahepatic biliary tract (Fig. 1a). Magnetic resonance cholangiopancreatography found a contracted gallbladder and measured the common bile duct as up to 4 mm in diameter without evidence of choledocholithiasis.

Upon admission, intravenous hydration and daily monitoring of relevant lab data were initiated. The course of LFT changes indicated a dramatic worsening of hepatotoxicity with an initial model for end‐stage liver disease (MELD) score of 24 shooting up to 38 over the course of 3 days. Also, international normalized ratio (INR) increased to 1.8 and the patient became confused, and asterixis appeared. Preparations for liver transplantation were made, and with the availability of an appropriate donor, orthotopic liver transplantation was performed 5 days after admission. Laparotomy revealed 3 L of clear ascites. A biopsy was performed during surgery, which revealed extensive confluent necrosis (Fig. 1b,c). There was no evidence of hepatic fibrosis or autoimmune hepatitis. Mild portal tract inflammation and scattered bile plugs were identified; however, pathologic findings did not point to a specific etiology. Post‐operative ultrasound revealed open hepatic veins, hepatic artery, and intrahepatic ducts with normal flow. Symptoms and lab data showed dramatic improvement after surgery, with jaundice remitting and bilirubin levels reducing dramatically from 38 to 4.6 mg/dL in 3 days. The patient's LFTs returned to normal levels within 8 days.

## Discussion

Here, we report a case of DILI caused by GA in a known case of MS in a 43‐year‐old woman referred to our center with jaundice, nausea, vomiting, abdominal pain, abnormal LFT, and impaired coagulation assays, which led to liver transplantation 10 weeks after the discontinuation of GA. Liver transplantation dramatically improved the signs, symptoms, and laboratory findings. To our knowledge, this is the first case of liver failure caused by GA that did not remit even after discontinuing GA and with common supportive treatments. Not only did liver function not return to normal, but also a very sharp increase in the LFT and INR in addition to a very high MELD score was observed, leading to the decision to perform a liver transplantation surgery.

The mechanism of functioning of GA, a synthetic analog of myelin basic protein in MS, is still unclear; however, modulating the inflammatory response as well as its neuroprotective and/or neuro‐regenerative effects are among the proposed mechanisms.^11^ GA has proved to be effective in reducing both the frequency of relapsing in addition to the burden and activity of MS.^12^ Our patient received GA as the second‐line treatment after the failure of corticosteroid therapy in the management of MS.

Twelve cases of liver damage caused by GA—including 8 DILI and 4 autoimmune hepatitis cases—have been reported in the literature.^10^ Neumann *et al*.^13^ reported the first case of liver injury induced by GA in 2007. The patient was a 71‐year‐old man who presented with jaundice and malaise secondary to GA in Germany. Viral markers were negative and the patient recovered 30 days after discontinuation of GA. The typical picture of a GA‐induced DILI case is a young woman presenting with jaundice, fatigue, and malaise with elevated LFT and bilirubin values and negative viral and autoimmunity markers who recovered 30–147 days after drug discontinuation.^10^ Although the precise way in which GA causes damage to liver tissue is not yet fully understood, the exacerbation of autoimmune conditions has been suggested as a possible mechanism.^13^ In patients with no evidence of prior autoimmune conditions, the observed patterns of mitochondrial changes have suggested mitochondrial inhibition as the key pathophysiological process, leading to elevated levels of reactive oxygen species and eventual cell death.^14^

In this case, the main point is that physicians, especially neurologists, should pay special attention to liver disorders caused by medications used in their medical regimens. It also emphasizes the special importance of comprehensive drug history and past medical history in a patient who has suffered from symptoms of acute liver failure, for internists and gastroenterologists. Although routine liver checkup is not recommended in patients who use GA, there is a need for more research on the liver toxicity caused by this medication.

### Informed consent

Written consent to participate was taken from the patient.
